# Polyphenols and Metabolites Enhance Survival in Rodents and Nematodes—Impact of Mitochondria

**DOI:** 10.3390/nu11081886

**Published:** 2019-08-13

**Authors:** Benjamin Dilberger, Maike Passon, Heike Asseburg, Carmina V. Silaidos, Fabian Schmitt, Tommy Schmiedl, Andreas Schieber, Gunter P. Eckert

**Affiliations:** 1 Laboratory for Nutrition in Prevention and Therapy, Institute of Nutritional Sciences, Biomedical Research Center Seltersberg (BFS), Justus-Liebig-University of Giessen, Schubertstr. 81, 35392 Giessen, Germany; 2 Institute of Nutritional and Food Sciences, Molecular Food Technology, University of Bonn, Endenicher Allee 19b, 53115 Bonn, Germany

**Keywords:** caenorhabditis elegans, mitochondria, longevity, polyphenol, respiration, protocatechuic acid

## Abstract

(1) Background: Polyphenols (PP) play an important role in the prevention of non-communicable diseases and may contribute to healthy aging. To investigate the molecular and cellular aspects of PP metabolites on longevity with a focus on mitochondrial function, we applied a pre-fermented mixture of polyphenols (Rechtsregulat^®^, RR) to rodents and nematodes. (2) Methods: The lifespans of Navar Medical Research Institute (NMRI) mice and *C. elegans* were recorded. The heat-stress resistance (37 °C) of *C. elegans* N2 was measured using nucleic staining. Respiration and membrane potential (ΔΨm) were measured in isolated mitochondria. The energetic metabolites adenosine triphosphate (ATP), lactate, and pyruvate were determined in lysates. Expression levels of longevity related genes were determined using quantitative real time polymerase chain reaction (qRT-PCR). Phenolic compounds were identified using ultra high performance liquid chromatography-diode array detection-Iontrap-multiple stage mass spectrometry (UHPLC-DAD-Iontrap-MS^n^). (3) Results: Several phenolic metabolites including protocatechuic acid (PCA) were identified in RR. Feeding of mice with RR resulted in a significantly increased lifespan. Heat-stress resistance (RR *** *p* = 0.0006; PCA **** *p* < 0.0001), median lifespan (NMRI: RR ** *p* = 0.0035; *C. elegans* RR * *p* = 0.0279; PCA **** *p* < 0.0001), and activity of mitochondrial respiratory chain complexes (RR *^−^** *p* = 0.0237 − 0.0052; PCA * *p* = 0.019 − 0.0208) of *C. elegans* were significantly increased after incubation with RR (10%) or PCA (780 µM). PCA significantly improved nematodes ΔΨm (* *p* = 0.02058) and ATP levels (* *p* = 0.029). RR significantly up-regulated lactate levels, indicating enhanced glycolysis. The expression levels of longevity related genes *daf-16*, *sir-2.1*, and *skn-1* were significantly upregulated after PCA, and partially after RR administration. (4) Conclusion: Phenolic metabolites such as PCA have the potential to enhance health and lifespan and mitochondrial function, and thus may contribute to healthy aging.

## 1. Introduction

Sociodemographic evaluations indicate a demographic shift towards older populations in developed as well as developing countries. As a result of an aging society, age-related diseases, such as cardiovascular, metabolic, and neurodegenerative diseases, have become more of a concern, making aging an important and worldwide topic [1,2]. Healthy aging depends on several factors, such as genetics and environmental factors. In particular, nutrition has proven to be a powerful tool to modulate aging [3].

Mitochondrial dysfunction has long been associated with aging and the development of age-related diseases [4,5]. A decline in mitochondrial function, and thus the loss of a sufficient energy production, supposedly plays key roles during aging. Therefore, developing ways to prevent mitochondrial dysfunction is a potent strategy to counteract adverse effects associated with aging [6,7,8,9].

In recent years physical activity and dietary patterns, such as the “Mediterranean diet” (MeDi), which is rich in polyphenols (PP), have become more important to stimulate the brain and mitochondrial capacity and increase overall health. These findings were confirmed by several recent meta-analyses [10,11,12]. In particular, secondary plant components such as PP were the focus of recent investigations. According to various reports, plant extracts containing a variety of the above-mentioned substances might have the same or an even stronger health-promoting effect than their single components [13]. However, several human intervention studies failed to show the anticipated beneficial effects of PP. The reasons for this are low bioavailability and extensive metabolism of PP. After ingestion, PP undergo a complex structural rearrangement through metabolism by the intestinal microbiota [14,15]. Thus, preclinical investigations on the molecular and cellular aspects of PP for biomedical questions should include metabolites of PP that in fact can reach the body´s bloodstream [14]. For our studies, we therefore select a fruit and vegetable extract rich in polyphenols (Rechtsregulat^®^, RR), pre-fermented by *Lactobacillus rhamnosus* and *L. casei* [16,17]. There is evidence that fermented foods provide health benefits well beyond the original food materials [18]. Fermentation causes changes in bioactive compounds and converts phenolic compounds to molecules with added biological value [19]. A recent analysis of RR identified flavonones such as naringenin and hesperetin, flavones such as luteolin and apigenin, and simple phenolic compounds with biological value such as protocatechuic acid (PCA) [20], which can be found in the human bloodstream and is believed to comprise potential beneficial effects [21]. PCA has antioxidative properties and shows positive effects on mitochondrial function and energy production [22,23].

However, a high performance liquid chromatography (HPLC) fingerprint revealed many un-identified phenolic compounds in RR [20]. Therefore, a state of the art technique including liquid chromatography-multiple stage mass spectrometry (LC-MS^n^) analysis with a linear iontrap was applied to further identify unknown components of RR. To obtain initial insights into how RR influences aging processes, the effects of RR and PCA [24], one of its major metabolites, on overall lifespan in mice and wild-type N2 *Caenorhabditis elegans* (*C. elegans*) were investigated. Moreover, the effects of RR and PCA on respiratory chain capacity, mitochondrial membrane potential (∆Ψm), energy production, and expression of genes involved in longevity, mitochondrial biogenesis, and function in the nematode model were studied.

## 2. Materials and Methods

### 2.1. Chemicals and RR

The chemicals used were of the highest available purity and standard from Sigma Aldrich (St. Louis, MO, USA) or Merck (Darmstadt, Germany). Rechtsregulat^®^ (RR) is a commercially available product derived from a stepwise (“cascade”) fermentation of 14 vegetables and fruits by different strains of *lactobacilli* according to the European Patent EP No 1153549 (11/17/04) [25]. The extract was provided from Dr. Niedermaier Pharma GmbH, Hohenbrunn, Germany. A HPLC fingerprint of Rechtsregulat^®^ was performed by Hippeli et al., which identified PCA as one of its major metabolites, with a concentration in the Regulat of 7.8 mM [20]. This concentration was adjusted to the applied concentration of 10% RR and chosen for all of the experiments. PCA was dissolved in ethanol and diluted to a working concentration of 1%, which is well tolerated by the organism [26]. Paraquat (PQ) served as the negative control and was dissolved in water to a working concentration of 2.5 or 5 mM, depending on the experiment.

The solvents and reagents used for liquid chromatography-mass spectrometry (LC-MS) analysis were purchased from the following suppliers. LC-MS-grade water, acetonitrile, and formic acid were obtained from ChemSolute (Renningen, Germany). HPLC-grade ethyl acetate was purchased from VWR International (Langenfeld, Germany). The 4-Hydroxybenzoic acid (99%), trans-cinnamic acid (99%+), and apigenin (97%) were from Alfa Aesar (Thermo Fisher Scientific, Kandel, Germany). Hesperidin (approx. 80%), hesperetin (analytical standard), naringenin (95%), *p*-coumaric acid (≥98.0% HPLC), 3,4-dihydroxyhydrocinnamic acid (98%), and quercetin (≥95.0% HPLC) were purchased from Sigma Aldrich (Steinheim, Germany). Luteolin (≥99%) and daidzein (≥99%) were obtained from Extrasynthese (Genay Cedex, France). Gallic acid (pro analysi > 98% HPLC) was from Fluka (Munich, Germany) and kaempferol (>99.0%) was from Phytoplan (Heidelberg, Germany).

### 2.2. Characterization of Phenolic Compounds in RR

#### 2.2.1. Sample Preparation

For the characterization of the phenolic composition of RR, the following procedure was applied: 1 mL RR was vortexed with 1 mL ethyl acetate and sonicated for 10 min. After centrifugation (5 min, 17,000× *g*, 20 °C), the ethyl acetate layer was collected and the extraction procedure was repeated twice with the residue. The combined ethyl acetate layers were reduced to one quarter of the initial volume using a nitrogen flow at 40 °C. The sample was filtered through a disposable filter (regenerated cellulose, pore size 0.20 µm, Macherey-Nagel GmbH and Co. KG, Düren Germany) and 5 µL was analyzed by UHPLC-DAD-Iontrap-MS^n^ (Thermo Scientific, Waltham, MA, USA).

#### 2.2.2. UHPLC and MS Settings

Analysis of the extracted RR was performed on an Acquity UHPLC system by Waters coupled with an LTQ XL linear ion trap by Thermo Scientific (Waltham, MA, USA). The Acquity UPLC consisted of a binary pump, an autosampler (cooled at 20 °C), a column oven set at 40 °C, and a diode array detector scanning from 190 to 700 nm. An Acquity HSS-T3 RP18 column (150 mm × 2.1 mm; 1.8 µm particle size) combined with a pre-column (Acquity UPLC HSS T3 VanGuard, 100 Å, 2.1 mm × 5 mm, 1.8 µm), both from Waters (Milford, MA, USA), were used for chromatographic separation with water (A) and acetonitrile (B) as eluents, both acidified with formic acid (0.1 + 99.9, *v* + *v*). The flow rate was set at 0.5 mL/min with a gradient elution: 0.0–1.5 min: 1.0% B (curve 6); 1.5–18.0 min: 1.0–25.0% B (curve 6); 18.0–30.0 min: 25.0–100% B (curve 6); 30.0–35.0 min: 100% B (curve 6); 35.0–40.0 min: 100–1% B (curve 3). A linear ion trap with an electrospray interface operating in negative and positive ion mode was used. The mass spectrometry (MS) settings were as follows: sheath gas flow: 70 arb; auxiliary gas flow: 10 arb; sweep gas flow: 1 arb; capillary temperature: 350 °C. Negative mode: source voltage: 3.00 kV; capillary voltage: −21.00 V; multipole 00 offset: +5.50 V; multipole 0 offset: +6.00 V; multipole 1 offset: +8.50 V; front lens: 6.50 V; positive mode: source voltage: 4.50 kV; capillary voltage: 11.00 V; multipole 00 offset: −7.75 V; multipole 0 offset: −8.25 V; multipole 1 offset: −12.50 V; front lens: −8.50 V. Normalized collision energy: MS^2^ 35%, MS^3^ 35%, using helium as collision gas. The system was controlled using Xcalibur Software 2.2 SP1.48 (Thermo Scientific, Waltham, MA, USA).

### 2.3. Nematode (Caenorhabditis elegans)

#### 2.3.1. *C. elegans* and Bacterial Strains

*C. elegans* wild-type strain N2 was obtained from the *Caenorhabditis elegans Genetics Center* (University of Minnesota, MN, USA). Nematodes were maintained on nematode growth medium (NGM) agar plates seeded with *E. coli* OP50 at 20 °C according to standard protocols [27]. For all experiments, synchronous populations were generated through a standard bleaching protocol [28].

#### 2.3.2. Cultivation and Treatment

Synchronous larvae were washed twice in M9 buffer, counted, and adjusted to 10 larvae per 10 µL. Depending on the experiment and on the number needed, nematodes were either raised in 96-well plates (Greiner Bio-One, Frickenhausen, Germany) or cell culture flasks (Sarstedt, Nürmbrecht, Germany). Concentration of OP50 was adjusted to 1 at optical density OD_600_ with liquid nematode growth medium (NGM). OP50-NGM was added as a standardized food source with a volume 4.4-fold of the larvae containing M9 solution used. L1 larvae were maintained under continuous shaking at 20 °C and reached adulthood within 3 days.

Effectors were added after reaching young adulthood 48 h prior to the experiment, with a final concentration of 5% or 10% RR, 2.5 mM or 5 mM PQ, and 780 µM PCA. Standard buffer M9 was used simultaneously as a control medium.

#### 2.3.3. Lifespan

To determine the lifespan of nematodes under physiological and standardized conditions (20 °C), a modified protocol from Amrit et al. was applied [29], and synchronized larvae obtained from egg preparation as mentioned above were raised on NGM agar plates spread with standard OP50 *E. coli* culture. After completing the L4 larval, stage 60 healthy animals per group were transferred to fresh NGM *E. coli* containing plates with a sterilized platinum wire. Effectors were incorporated into the OP50 culture at the necessary concentration. To distinguish adult nematodes of the starting population from freshly laid eggs, the plates were checked every 48 h and adults transferred to new effector-containing plates (after nematodes stopped producing progeny, they were no longer transferred to new plates but still checked every 48 h for life signs). In line with the separation from eggs and larvae, nematodes were checked for vital signs using a hot platinum wire held next to the animals’ heads. Worms showing no reaction to the heat stimulus were considered dead. Nematodes that crawled off the plates or were killed while being transferred were censored from the experiment. The survival curves were compared statistically using the log-rank test.

#### 2.3.4. Heat-stress Resistance

Approximately 10 nematodes were raised per well in a 96-well microplate, as mentioned above. After 48 h of incubation effectors were given, as previously described. Time until death of the nematodes was determined using a microplate thermo-tolerance assay, as described by Fitzenberger et al. [30]. In brief, nematodes were washed off the wells with M9 buffer into 15 mL tubes followed by three additional washing steps. In each well of a black 384-well low-volume microtiter plate (Greiner Bio-One, Frickenhausen, Germany), 6.5 μL M9 buffer/Tween^®^ 20 (1% *v*/*v*) solution were added. Subsequently, 1 μL M9 buffer containing one nematode was dispensed into each well under a stereomicroscope (Breukhoven Microscope Systems, Capelle aan den Ijssel, Netherlands), and mixed with 7.5 μL SYTOX™ green (final concentration 1 μM; Life Technologies, Karlsruhe, Germany). To prevent water evaporation, the plates were sealed with a Rotilab sealing film (Greiner Bio-One, Frickenhausen, Germany). Heat shock (37 °C) was induced and fluorescence was measured with a ClarioStar plate reader (BMG, Ortenberg, Germany) every 30 min over the course of 17 h. To detect SYTOX™ green fluorescence, the excitation wavelength was set at 485 nm and the emission detected at 538 nm.

#### 2.3.5. Quantitative Real-time PCR

Total RNA was isolated using the RNeasy Mini Kit (Quiagen, Hilden, Germany) according to the manufacturer´s instructions. After collecting 4000 nematodes, their cuticles were disrupted using a Balch Homogenizer with a 10 µM Clearance. The concentration of RNA was quantified by measuring the absorbance at 260 and 280 nm using a NanoDrop™ 2000c spectrometer (Thermo Fisher Scientific, Waltham, MA, USA). RNA purity was assessed using the ratios of absorbance 260/280 and 260/230. Afterwards, samples were treated with a TURBO DNAfree kit™ (Thermo Fisher Scientific, Waltham, MA, USA) to remove residual genomic DNA. According to the manufacturer´s guidelines, complementary DNA was synthesized from 1000 µg total RNA using an iScript cDNA Synthesis Kit (Bio-Rad, Munich, Germany) and temporarily stored at −80°C. The qRT-PCR was conducted using a CfX 96 Connect™ system (Bio-Rad, Munich, Germany). Primers were obtained from Biomers (Ulm, Germany). Oligonucleotide primer sequences, primer concentrations, and product sizes are listed in Table 1. All cDNA samples were measured in triplicate after a 1:10 dilution with RNase-free water (Quiagen, Hilden, Germany). PCR cycling conditions were conducted as follows. An initial denaturation at 95 °C for 3 min, followed by 45 cycles of 95 °C for 10 s, 58 °C for 45 s, and 72 °C for 29 s. Gene expression was analyzed using the –(2ΔΔC_q_) method with BioRad CfX manager software and normalized to the expression levels of amanitin resistant (*ama-1*) and actin (*act-2*).

#### 2.3.6. Isolation of Mitochondria

To isolate functional mitochondria, a very precisely calculated population of 5000–10,000 adult nematodes, depending on the experiment, per group was required. Nematodes were raised in liquid NGM culture and incubated with effectors, as stated above under standardized conditions. 

Two-day-old gravid adults were separated from larvae using a self-made separation device with a nylon mesh (Dr. Fill^®^, Giessen, Germany), and washed several times with M9 buffer to remove bacteria and debris, and finally transferred to ice-cold isolation buffer (300 mM Sucrose, 5 mM 2-[(2-hydroxy-1,1-bis(hydroxymethyl)ethyl)amino]ethanesulfonic acid (TES), 200 µM ethylene glycol-bis(2-aminoethylether)-N,N,N′,N′-tetraacetic acid (EGTA), pH 7.2) as described by Schmitt et. al. [31].

To obtain a mitochondria-enriched fraction, a Balch Homogenizer (Isobiotec, Heidelberg, Germany) was used [32]. Nematodes were gently passed through the homogenizer a total of 5 times with 1 mL glass syringes (SGE Syringe, Trajan, Australia) fitted with a metal luer lock to break open and homogenize the animals; a 12 µm ball clearance was applied. The homogenate was centrifuged at 800 g for 5 min at 4 °C (Haraeus Fresco 21, Thermo Scientific, Langenselbold, Germany) to discard debris and larger worm breakage. The mitochondria containing supernatant was collected and centrifuged at 9000× *g* for 10 min at 4 °C. The crude mitochondria containing pellet was resuspended in 70 µL swelling buffer (SWB) (0.2 M sucrose, 10 mM MOPS-Tris, 5 mM succinat, 1 mM H_3_PO_4_, 10 µM EGTA, 2 µM rotenone) for membrane potential (ΔΨm) experiments [31] or 200 µL of mitochondrial respiration medium MirO5 (0.5 mM EGTA, 3 mM MgCl_2_, 60 mM K-lactobionate, 20 mM taurine, 10 mM KH_2_PO_4_, 20 mM HEPES, 110 mM sucrose, 1 g/L BSA, pH 7.1; developed by Oroboros) for high-resolution respiratory experiments [33]. For ΔΨm and respiration measurements, fresh mitochondria were immediately used. Spare samples were shock frozen in liquid nitrogen for determination of citrate synthase activity and protein content.

#### 2.3.7. High-resolution Respirometry

Respiration experiments were conducted at 20 °C using a Clark-type electrode (O2k Oxygraph, Oroboros Instruments, Innsbruck, Austria). For each measurement an aliquot of 80 µL in MirO5 resuspended mitochondria, as described previously, was inserted into 2 mL of air-saturated MirO5-containing electrode chamber.

For analysis, the provided DatLab software (Version 7.0.0.2, Oroboros Instruments, Innsbruck, Austria) was used.

To determine mitochondrial function a complex protocol (Oroboros Instruments, Innsbruck, Austria) was applied, as previously stated [34]

Mitochondrial respiration was normalized to citrate synthase activity.

#### 2.3.8. Mitochondrial Membrane Potential (ΔΨm)

To determine the mitochondrial membrane potential, a modified protocol described by Zischka et al. was applied [31]. A total of 5000 nematodes was needed to obtain an adequate mitochondrial pellet following the described Balch homogenization using a 12 µm clearance. The fluorescent dye Rhodamine 123 (Rh123) was used to assess the ΔΨm of in 25 µL SWB isolated mitochondria in a black 96-well plate with a ClarioStar plate reader (BMG, Ortenberg, Germany). To ensure mitochondrial integrity, the potential was measured for 30 minutes and if steady carbonyl cyanide 4-(trifluoromethoxy) phenylhydrazone (FCCP) (500 nM) was added to evaluate the membrane specific quenching effect of Rh123. Results were normalized to the number of nematodes.

#### 2.3.9. Citrate-Synthase Activity

As previously stated an aliquot of 70 µL in MirO_5_ isolated mitochondria was shock frozen in liquid nitrogen for determination of citrate synthase activity. After thawing, a reaction mix containing 0.5 mM oxaloacetate, 0.1 mM DTNB, 0.31 mM acetyl coenzyme A, 50 µM EDTA, 5 mM triethanolamine hydrochloride, and 0.1 M Tris-HCl was prepared and pre-heated for 5 minutes at 30 °C. To determine citrate synthase (CS) activity, 10 µL of mitochondria suspension was added and assessed spectrophotometrically at 412 nm [35,36]. Measurements were performed in triplicate.

#### 2.3.10. Nematode Homogenization

For determination of the energetic metabolites ATP, lactate, and pyruvate, a nematode homogenate was generated. In brief, 4000 synchronized nematodes were harvested, thoroughly washed, shock frozen, and boiled for 15 min prior to sonification to denaturate degrading proteins. After centrifugation at 15000× *g* for 10 min, supernatants were collected. ATP content was assessed immediately, and spare samples were stored at -80 °C for later determination of lactate, pyruvate, and protein content.

#### 2.3.11. Determination of ATP

Intracellular ATP levels were determined using the ATPlite (Perkin Elmer, Waltham, MA, USA). Luminescence was measured in triplicate following the manufacturer´s guidelines using a ClarioStar plate reader (BMG, Ortenberg, Germany). Spare samples were frozen at -80 °C for determination of protein content.

#### 2.3.12. Determination of Lactate and Pyruvate Content

Frozen homogenate samples slowly thawed until they reached room temperature. Concentrations of lactate and pyruvate were assessed using two colorimetric assay kits from Sigma (MAK064 and MAK071) following the manufacturer´s guidelines (Sigma Aldrich, St. Louis, MO, USA) using a ClarioStar plate reader (BMG, Ortenberg, Germany).

#### 2.3.13. Protein Quantification

Protein contents were assessed according to the Pierce™ BCA Protein Assay Kit (Thermo Fisher Scientific, Waltham, MA, USA). Bovine serum albumin was used as standard.

### 2.4. Rodents 

#### 2.4.1. Animals and Treatment

Navar Medical Research Institute (NMRI) mice, a commonly used model for aging, were purchased from Charles River Laboratories (Sulzbach, Germany) and housed according to the German guidelines for animal care with ad libitum access to food and water. The mice were maintained in a 12 h light/dark cycle. A total of 44 male and female NMRI mice were divided into two groups of the same distribution. A control group was served with regular tab water, whereas the treatment group was given RR (10%) starting after the 20th month of age. All of the experiments were carried out by individuals with appropriate training and experience according to the requirements of the Federation of European Laboratory Animal Science Association and the European Communities Council Directive (Directive 2020/63/EU). Experiments were approved by the regional authorities.

#### 2.4.2. Survival

For the survival experiments mice were allowed to live out their lives. Once a day every mouse was checked for vital signs, and thus the time of death was determined. The survival curves were compared statistically using the log-rank test.

### 2.5. Statistics

Unless otherwise stated, values are presented as mean ± standard error of means (SEM). Statistical analyses were performed by applying one-way Analysis of Variance (ANOVA) with Tukey´s multiple comparison post-test (Prism 8.0 GraphPad Software, San Diego, CA, USA). Statistical significance was defined for *p* values * *p* < 0.05, ** *p* < 0.01, *** *p* < 0.001, and **** *p* < 0.0001.

## 3. Results

### 3.1. Characterization of Phenolic Compounds in RR

**Figure 1:**
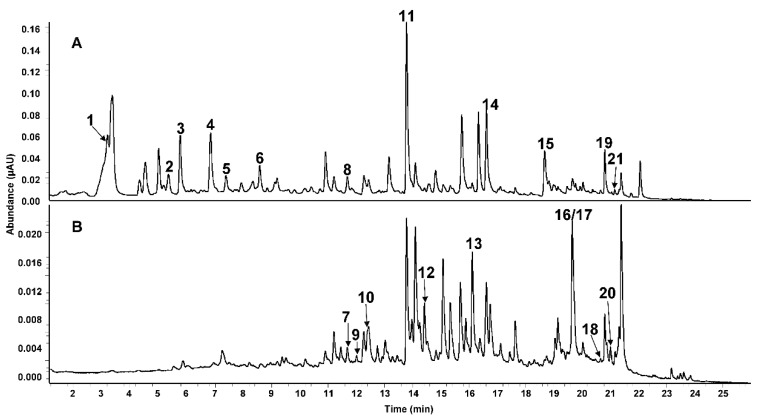
UHPLC chromatograms of RR. (**A**) 280 nm; (**B**) 350 nm. For peak numbers see Table 2.

For the characterization of phenolic compounds, their UV-spectra, MS^n^-spectra, and chromatographic behavior were compared to published data and to reference compounds where available. In total, 21 phenolic compounds were detected, among them six phenolic acids, four flavanones, five flavones, four flavonols, one isoflavone, and one trihydroxybenzene. Retention times, UV maxima, and MS^n^ data are shown in Table 2. Typical UHPLC chromatograms are shown in Figure 1.

#### 3.1.1. Hydroxybenzoic and Hydroxycinnamic Acids

In RR three hydroxybenzoic and three hydroxycinnamic acids were present. The identification of gallic acid (**1**), protocatechuic acid (**2**), 4-hydroxybenzoic acid (**5**), 3,4-dihydroxyhydrocinnamic acid (**6**), and *p*-coumaric acid (**8**) was based on the comparison of retention times, UV-spectra, and MS^n^ fragmentation patterns with those of reference substances (Table 2). Additionally, a second dihydroxyhydrocinnamic acid (**4**) was detected, which exhibited a molecular ion mass of *m*/*z* 181. The assignment was corroborated by its fragmentation pattern, since in the *MS*^2^ spectra product ions at *m*/*z* 163 [M−H−18]^−^ and *m*/*z* 135 [M−H−46]^−^ were observed. However, the exact position of the two hydroxyl groups cannot be assigned solely on the basis of mass spectrometry.

#### 3.1.2. Flavanones

Hesperetin-7-*O*-rutinoside (hesperidin, **14**), naringenin (**18**), and hesperetin (**21**) were tentatively identified on the basis of reference compounds. Eriodictyol-7-*O*-rutinoside (eriocitrin, **11**) exhibited a molecular mass [M−H]^−^ at *m*/*z* 595 and the fragmentation led to the product ion *m*/*z* 287 in the MS^2^ spectra, indicating the loss of the rutinose moiety. Consecutive fragmentation of *m*/*z* 287 yielded the ^1,3^A^−^ ion as the base peak at *m*/*z* 151, which is typical for the aglycone eriodictyol.

#### 3.1.3. Flavones

The two flavone aglycones, luteolin (**16**) and apigenin (**19**), were identified by means of the comparison of retention time, UV-spectra and MS^n^ fragmentation pattern with the reference substances available. The molecular mass of compound **7** at *m*/*z* 593 yielded product ions at *m*/*z* 473, 503, 353, and 575, which corresponds to a loss of [M−H−120]^−^, [M−H−90]^−^, [M−H−240]^−^, and [M−H−18]^−^, respectively. Consecutive fragmentation of *m*/*z* 473 exhibited a further loss of 120 Da (*m*/*z* 353) and 90 Da (*m*/*z* 383), indicating the ring cleavage of a *C*-bound hexose moiety. Thus, Compound **7** was tentatively identified as apigenin-6,8-di-*C*-glucoside (vicenin-2). Furthermore, Compounds **9** and **10** exhibited both a molecular mass [M−H]^−^ at *m*/*z* 623 and in the MS^2^-spectra the loss of H_2_O [M−H−18]^−^ as well as the [M−H−120]^−^, [M−H−90]^−^, and [M−H−240]^−^ fragments, indicating the presence of a *C*-hexoside. The fragmentation patterns of both compounds were identical. Based on this data, Compounds **9** and **10** were tentatively identified as luteolin-6,8-di-*C*-glycoside methyl ether isomers (lucenin-2 methyl ether isomers).

#### 3.1.4. Flavonols

The comparison of the chromatographic and mass spectrometric behavior of Compounds **17** and **20** with reference compounds led to the identification of quercetin (**17**) and kaempferol (**20**). In addition, two further flavonols with a typical UV maximum at 350 nm were detected at *m*/*z* 447 (**13**) and *m*/*z* 593 (**12**), respectively. The fragmentation of *m*/*z* 447 exhibited the product ion *m*/*z* 301, indicating the loss of a deoxy sugar [M−H−146]^−^. The quercetin aglycone was present after the typical homo- and heterolytic cleavage at *m*/*z* 300 and *m*/*z* 301. Further fragmentation of the product ion *m*/*z* 301 generated characteristic fragment ions in the MS^3^ spectra of quercetin at *m*/*z* 179, 151, and 273, which corresponds to the ions ^1,2^A^−^, ^1,2^A^—^CO, and [M−H−CO]^−^. Hence, Compound **13** was tentatively identified as quercetin rhamnoside. The observed loss of [M−H−308]^−^ is characteristic of a disaccharide moiety, such as rutinose, and produced the product ion *m*/*z* 285, further fragmentation behavior of which (see Table 1) indicated the kaempferol aglycone. Thus, Compound **12** was tentatively identified as kaempferol rutinoside. 

#### 3.1.5. Further Phenolic Compounds

One isoflavone and one trihydroxybenzene were tentatively identified in RR. Compound **15** was identified as the isoflavone daidzein, based on the comparison of retention time, UV maximum, and MS fragmentation patterns with a reference substance. In the positive ionization mode, the molecular mass [M+H]^+^ at *m*/*z* 127 yielded the product ion *m*/*z* 109, which corresponds to a loss of H_2_O [M+H−H_2_O]^+^, and therefore Compound **3** was tentatively identified as pyrogallol.

### 3.2. Effect on Lifespan in Mice and Nematodes

#### 3.2.1. Physiological Lifespan

**Figure 2:**
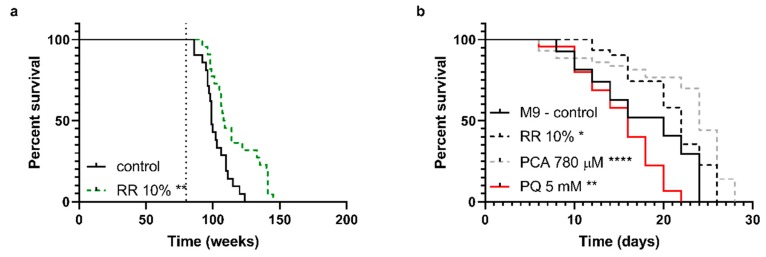
(**a**) Survival rates of NMRI mice with or without supplementation of RR via drinking water until time of death. Mice received a 10% supplementation of RR starting with the age of 20 months (indicated by the dotted line), leading to a significantly increased lifespan. (**b**) Survival of wild-type *C. elegans* N2 treated with RR 10%, PCA 780 µM, and paraquat 5 mM was determined under physiological conditions until day of death; *n* = 22 (a; NMRI), *n* = 48 (b; C57BL/6J), and *n* = 27–44 (c; N2); log-rank (Mantel-cox) test; * *p* < 0.05, ** *p* < 0.01, *** *p* < 0.001, and **** *p* < 0.0001.

NMRI mice (Figure 2a), a common mouse model for aging studies, were fed with a standardized pelleted diet *ad libitum* and tab water mixed with RR for the intervention group after reaching 20-months of age (indicated by dotted line in Figure 2a), as stated above. RR increased the median survival of NMRI mice significantly from 99 to 108.5 days (** *p* = 0.0035). 

For *C. elegans,* the median survival improved significantly by ten percent after incubation with 10% RR (median = 22 days; * *p* = 0.0279) and 20% with 780 µM PCA (median = 24 days; **** *p* < 0.0001), respectively. PQ (5 mM) significantly reduced the animals median lifespan by 20% (median = 16 days; ** *p* = 0.0024) compared to M9 control (median = 20 days) (Figure 2b).

#### 3.2.2. Heat-stress Resistance

**Figure 3:**
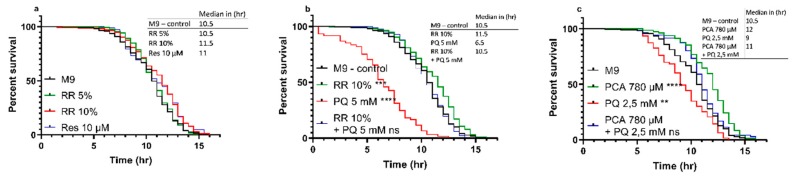
Heat-stress resistance of *C. elegans* at 37 °C under the influence of RR 5 and 10%, PCA 780 µM, paraquat 2,5 and 5 mM, and combinations of said effectors over the course of 17 h (**a–c**). For heat-stress experiments, survival was assessed according to the penetration of SYTOX™ Green nucleic acid stain into dead cells, as previously described, with a minimum of *n* < 61; log-rank (Mantel-Cox) test; ** *p* < 0.01, *** *p* < 0.001, and **** *p* < 0.0001.

*C. elegans* showed improved heat-stress resistance after treatment with RR and PCA in a dose-dependent manner. While for 5% RR, only a non-significant numerical (median = 10.5 h; *p* = 0.4740) shift to the right of the survival curve could be observed, a dose of 10% was able to significantly improve the heat-stress resistance (median = 11.5 h; *** *p* = 0.0006). The effect of RR (10%) was numerically but not significantly bigger than 10 µM resveratrol (median = 11 h; ** *p* = 0.0068) compared to M9 control (median = 10.5 h) (Figure 3a). At a concentration of 10%, RR was also able to negate the highly significant decrease in heat-stress resistance induced via insult with mitochondrial stressor paraquat (PQ) at a concentration of 5 mM (median = 6.5 h; **** *p* < 0.0001) back to the level of the M9 control group (median = 10.5 h; *p* < 0.6366) (Figure 3b).

A similar result could be shown for protocatechuic acid (PCA). A concentration of 780 µM PCA (equal to the concentration in 10% RR) improved the nematodes heat-stress resistance significantly (median = 12 h; **** *p* < 0.0001) compared to M9 control (median = 10.5 h), whereas a 2.5 mM paraquat insult again caused a decreased heat-stress resistance (median = 9 h; ** *p* = 0.0015). As depicted in Figure 3b, 5 mM PQ severely reduced the survival of *C. elegans*. To access the effects of PCA in a milder stress condition, we therefore decided to reduce the PQ concentration to 2.5 mM. PCA was able to reverse the adverse effects of PQ and to ameliorate the nematodes stress resistance above the level of controls (median = 11 h; *p* = 0.0948) (Figure 3c). 

### 3.3. Quantitatice real-time PCR

**Figure 4:**
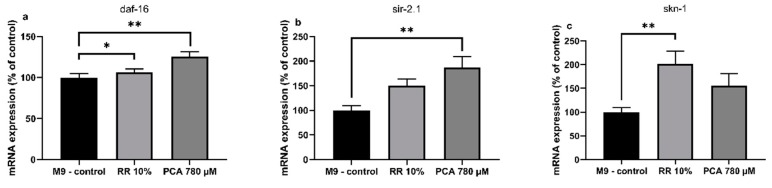
Relative normalized mRNA expression levels of longevity related genes (**a**) *daf-16*, (**b**) *sir-2.1* and (**c**) *skn-1* in *C. elegans after* treatment with RR (10%) and PCA (780 µM); mRNA expression of M9-control is 100%; *n* = 10; mean ± SEM; one-way ANOVA with Tukey´s multiple comparison posttest; * *p* < 0.05, ** *p* < 0.01; results are normalized to the mRNA expression levels of amanitin resistant (*ama-1*) and actin (*act-2*).

RR and PCA affected the expression levels of several longevity related genes in *C. elegans*. Expression of human forkhead box O1 ortholog *daf-16* (abnormal DAuer Formation) showed a non-significant numerical increase by RR (106% ± 5%; *p* = 0.6323) and a significant increase by PCA (125% ± 6%; ** *p* = 0.0063). An ortholog of human Sirtuin 1, *sir-2.1* (yeast SIRtuin related), was numerically increased by RR (150% ± 14%; *p* = 0.1049) and significantly by PCA (187% ± 23%; ** *p* = 0.0027). RR also elevated expression levels of *skn-1* (SKiNhead) significantly (201% ± 27%; ** *p* = 0.0065), while PCA treatment only led to a numerical increase (156% ± 23%; *p* = 0.2053) (Figure 4a–c).

### 3.4. Mitochondrial Function 

**Figure 5:**
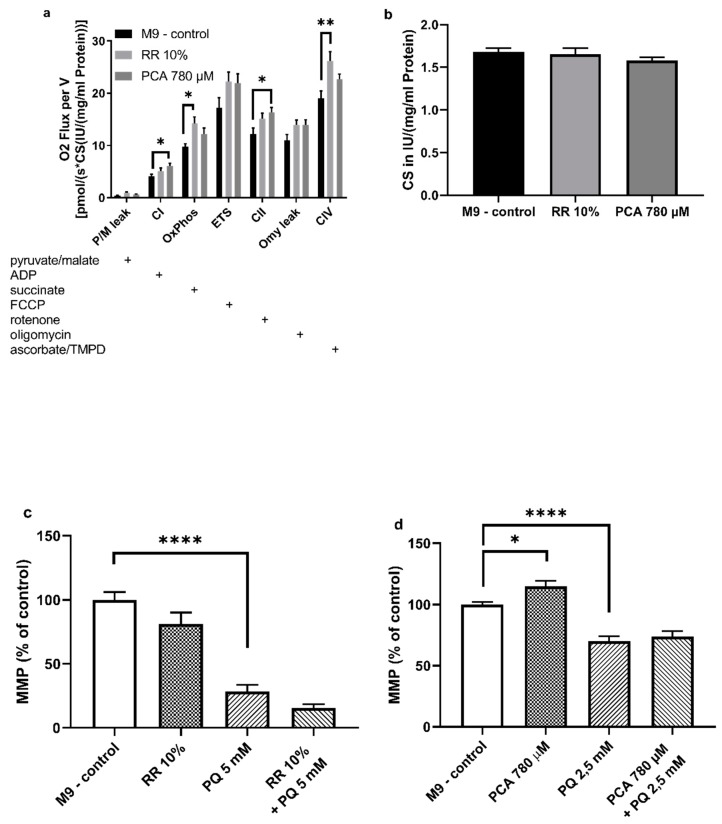
(**a**) Respiration of mitochondria isolated from *Caenorhabditis elegans* normalized to (**b**) citrate synthase activity in international units IU/(mg/mL protein). Activity of respiration complexes was measured using an O2k Oxygraph (Oroboros Instruments, Innsbruck, Austria). Addition of substances into the Oxygraph´s chambers is indicated with a plus sign. (**c**,**d**) Mitochondrial membrane potential *ΔΨm* assessed after addition of fluorescent dye Rhodamine 123 (Rh123) detected with a ClarioStar plate reader (BMG, Ortenberg, Germany); *n* = 7–13; mean ± SEM; one-way ANOVA with Tukey´s multiple comparison post-test; * *p* < 0.05, ** *p* < 0.01 and **** *p* < 0.0001.

In order to assess the effects of both substances on mitochondrial function, mitochondria of nematodes fed with the effectors mentioned above were isolated as described and measured with an O2k Oxygraph (Oroboros, Innsbruck, Austria). RR and PCA increased all complexes of the mitochondrial respiration chain, at least numerically. The activities of combined respiratory system complexes CI + CII (OXPHOS) (* *p* < 0.0237) and complex IV (** *p* < 0.0052) were significantly increased by RR. In contrast, PCA significantly increased CI (* *p* < 0.019) and CII (* *p* < 0.0208) respiration (Figure 5a). 

To determine mitochondrial content the activity of citrate synthase (CS) was measured [37]. No significant differences were observed after administration of RR and PCA (Figure 5b), indicating that no changes in mitochondrial content occurred [38].

The impact on mitochondrial membrane potential (ΔΨm) of RR and PCA was assessed using the fluorescent dye Rhodamine 123. RR (81% ± 24%; *p* = 0.2085) did not improve the ΔΨm of isolated *C. elegans* mitochondria compared to M9 control (100% ± 16%). In comparison to control, an insult with 5 mM paraquat resulted in a significant decrease of ΔΨm (30% ± 17%; **** *p* < 0.0001). The effects of this insult could not be compensated for by RR (16% ± 7%; *p* = 0.4408) (Figure 5c).

In contrast, PCA significantly increased ΔΨm (115% ± 15%; * *p* = 0.0258) compared to M9 control (100% ± 7%). Again, PQ (2.5 mM) significantly decreased ΔΨm (68% ± 12%; **** *p* < 0.0001) compared to M9 control (100% ± 7%). Although not significant, PCA was able to numerically attenuate the decrease in membrane potential after PQ insult (76% ± 14%; *p* = 0.4763) (Figure 5d).

**Figure 6:**
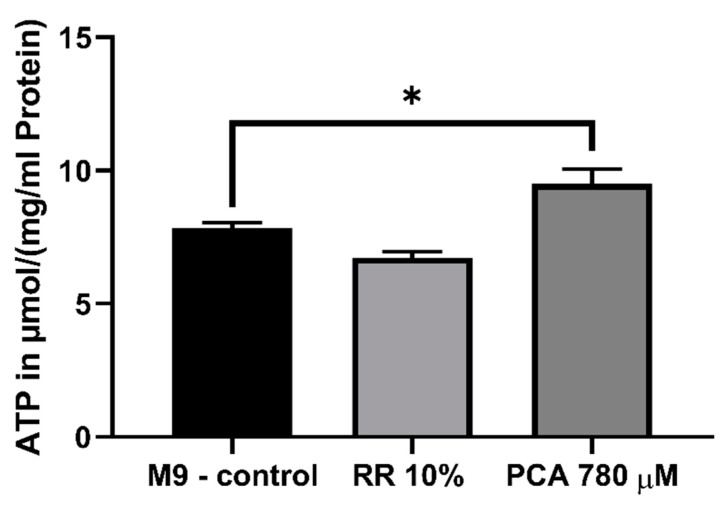
Determination of intracellular ATP levels in wild-type *C. elegans*. Animals were shock frozen and boiled for de-proteinization prior to cell lysation via sonification. ATP levels were assessed using ATPlite luminescence assay (Perkin Elmer, Waltham, MA, USA) and values normalized to protein concentrations. PCA increased levels significantly. *n* = 8; mean ± SEM; one-way ANOVA with Tukey´s multiple comparison posttest; * *p* < 0.05.

Since PCA significantly improved ΔΨm and alterations of the respiratory chain could be detected after treatment with RR as well as PCA. We next determined the mitochondrial energy end-product ATP. While treatment with 10% RR showed no significant changes in the ATP concentrations (*p* = 0.2094), the ATP concentration was significantly increased by 780 µM PCA (121%; * *p* = 0.029) (Figure 6).

### 3.5. Glycolysis

**Figure 7:**
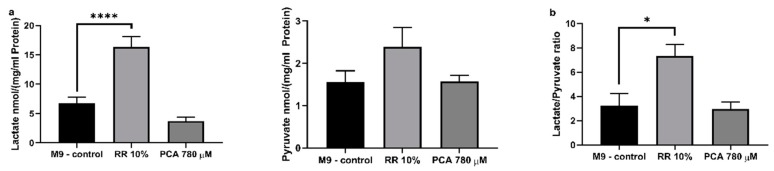
**(a)** Assessment of intracellular lactate and pyruvate concentrations in nematode homogenate and (**b**) the consequential lactate/pyruvate ratio. Levels were determined using two colorimetric lactate and pyruvate assay kits from Sigma (Sigma Aldrich, St. Louis, MO, USA). Values were normalized to protein concentrations; *n* = 8; mean ± SEM; one-way ANOVA with Tukey’s multiple comparison posttest; * *p* < 0.05 and **** *p* < 0.0001.

Beside mitochondrial respiration, cells also produce ATP by glycolysis. RR significantly increased intracellular lactate levels up to 243% (**** *p* < 0.0001; due to several washing steps and experimental procedures contamination of nematode homogenate with lactic acid through RR can be excluded) in contrast to PCA treatment, which had no significant effect. Both effectors showed no effects on pyruvate levels (Figure 7a). Consequently, the lactate/pyruvate ratio (Figure 7b) was significantly increased by 228% after RR treatment (** *p* = 0.0102).

## 4. Discussion

NMRI mice and the nematode *Caenorhabditis elegans* represent well-established and commonly used models for aging studies and the determination of mitochondrial function [30,39,40,41,42]. In this study, we tested the effect of 10% Rechtsregulat^®^ (RR) and one of its major components protocatechuic acid (PCA) on the lifespan of a common mouse model and wild-type N2 *C. elegans*, as well as mitochondrial parameters of the nematodes. Since RR represents a complex mixture of polyphenolic compounds we also investigated PCA, a well-known metabolite of polyphenols, such as hesperidin or quercetin [43], in a concentration equal to 10% RR [20]. PCA has a good bioavailability, crosses the blood-brain barrier, and has already been shown to have antioxidative and anticancerogenic activities [21]. Moreover, PCA shares structural similarities with well-documented polyphenols, such as caffeic acid, gallic acid, coumaric acid, vanillic acid, and ferulic acid [9,44]. 

In nematodes, heat-stress resistance, mitochondrial parameters, and energy metabolites, including ATP, lactate, and pyruvate levels, were investigated.

RR is rich in polyphenols and secondary plant metabolites, fermented by different strains of *Lactobacilli*. Polyphenols such as resveratrol or protocatechuic acid are well-known for their antioxidant, anti-inflammatory, antiapoptotic, anticancerogenic, and mitochondria-modulating capabilities, and thus have been studied frequently in the context of aging and mitochondrial function [39,44,45,46,47,48,49].

We used PQ, a compound formerly used as a herbicide, as a mitochondrial stressor to simulate mitochondrial function under pathological conditions. Even though its mechanism is not fully understood, recent findings suggest that PQ primarily targets complex CI and partially complex CII of the mitochondrial respiratory chain, resulting in oxidative stress [50,51]. This adverse effect can also be seen in our heat-stress, lifespan, and mitochondrial membrane potential experiments.

### 4.1. RR and PCA Improve Life and Health-span

RR prolonged the median survival rate of treated NMRI mice significantly from 99 days of controls to 108.5 days. This is in line with previously conducted studies that also investigated the positive effect of polyphenol rich diets in rodents on overall lifespan [39,49,52,53].

The nematode *C. elegans* was used to assess the molecular and cellular mechanisms of RR and PCA on longevity with a focus on mitochondrial function. A comparable extension in median lifespan was demonstrated for RR in *C. elegans* with an increase of 10% and an even stronger increase of 20% for PCA. These observed effects have already been well-documented in *C. elegans* for PCA [54] and other polyphenols, such as epicatechine and catechine [55], eppigallocatechin gallate [56], or resveratrol [57].

The major advantage for aging studies in *C. elegans*, compared to rodents, is their shorter lifespan, at approximately 30 days under physiological conditions. Various designs have been developed to study longevity with nematodes, including physiological aging at 20 °C and heat-stress resistance assays at, for example, 37 °C, as also conducted for this investigation. Although both designs investigate the animals time-point of death, there are distinct differences [58]. Compared to the straight forward lifespan design, in which worms are checked every two days for vital signs [29], heat-stress models focus on other aspects of aging. High temperatures cause neuronal degeneration and necrotic cell death in *C. elegans* regulated through forkhead box O (FOXO) transcription factors *daf-16* and heat-shock transcription factor-1 (*hsf-1*) in *C. elegans* [59,60]. Survival assays using *C. elegans* have been a crucial tool for unravelling mechanisms of aging, with an emphasis on neuronal decline, over recent decades. Thus, applying different designs focusing on different points of view of aging appears valid and crucial [60].

Overall, we demonstrated that PCA, as well as RR, were able to increase the nematodes heat-resistance significantly in a concentration-dependent manner, thus making it a potential tool to counteract age-related adverse effects, such as neuronal decline [58]. For both 10% RR and PCA, the positive effects were even stronger than those of resveratrol, which represents one of the most prominent polyphenols in this regard [57]. 

Several other polyphenols and polyphenol compositions have been investigated for their capacities to increase health-span. Fitzenberger et al. showed protective effects of quercetin against glucose-induced stress in a similar assay to the one used for this study [30]. A similar improved heat-stress resistance was shown for caffeic- and rosmarinic acid [61] and epigallocatechin-3-gallate, a major polyphenol in green tea [56]. While positive effects on longevity were demonstrated for grape skin extract in mice [39], comparable effects have been reported for blueberry [62] and cranberry extracts in *C. elegans* [63].

Mechanistically, it has been well documented that longevity and improved stress resistance is transmitted via *daf-16* in *C. elegans*. Saier et al. identified the forkhead box O1 (FOXO1) ortholog to be responsible for extensions in life- and health-span that were comparable to our own results by administration of *Agrimonia procera*, a herb rich in phytochemicals such as polyphenols [64]. The ortholog of Sirtuin 1 in *C. elegans, sir-2.1*, has been shown to regulate aging in the nematode via transcription factor *daf-16* [65]. Thus, activation of *sir-2.1,* in line with *daf-16,* appears feasible (see Figure 4a,b) and is coherent with our findings, which show significant increases in *sir-2.1* and *daf-16* expressions, as well as life- and health-span experiments after PCA and numerical improvements after RR treatment. Activation of transcription factors *daf-16* and *sir-2.1* have also been described for epigallocatechin-3-gallate [66], resveratrol and oxyresveratrol [67], and quercetin, caffeic-, and rosmarinic acids [61]. Although *sir-2.1* was not affected by chlorogenic acid, a polyphenol commonly found in coffee and tea, activity of *daf-16* and *skn-1* could be significantly increased [68]. *Caenorhabditis elegans* Nrf2 (nuclear factor E2 related factor 2) ortholog *skn-1*, which is responsible for phase II detoxification in response to oxidative stress [69], was also significantly increased after RR administration and numerically by PCA. This is not uncommon for substances with antioxidative activities and can also be seen for *Glycyrrhiza uralensis* [70] and cucurmin [71].

### 4.2. Ameliorated Mitochondrial Function Through RR and PCA

Mitochondrial dysfunction appears to be a hallmark of aging and age-related diseases and is hypothesized to play a major role in the early onset development of neurodegenerative diseases, such as Morbus Alzheimer [72,73,74]. Since RR and PCA show life-prolonging effects, we were interested in their influence on the mitochondrial respiratory chain system (see Figure 5a).

RR administration increased mitochondrial respiration in general, but the effect on oxidative phosphorylation (OXPHOS) (combined complex I + II) and CIV was most distinct. These results are very similar to the results of three recent studies investigating the effect of another extract rich in polyphenols. Here, a rice bran extract (RBE) was also able to increase OXPHOS and CIV respiration [49], as well as mitochondrial content in NMRI mice [75] and guinea pigs [34]. It is of note that our results for citrate synthase activity measurements, as a marker for mitochondrial mass [37], do not support the increased mitochondrial content ameliorated by RBE [34,75], indicating that increased OXPHOS activity is responsible for the increased respiration and not increased mitochondrial content.

In contrast, even though all complexes showed a numerically increased respiration rate, PCA improved CI and CII activity significantly. To our best knowledge the influence of PCA on the respiratory chain in isolated mitochondria with a Clarke-type electrode has never been tested before, but various other polyphenols, such as curcumin, quercetin, or resveratrol, have shown beneficial effects on mitochondrial function [76].

In mammals, PGC1α is a key regulator of mitochondrial biogenesis [77]. *C. elegans,* however, does not have a homolog with a similar function. Biogenesis and mitochondrial turn-over, at least in part, are regulated in nematodes via *skn-1* an ortholog of mammalian Nrf2 [78], and *sir-2.1,* which controls *daf-16* [79,80], which links mitochondrial function and longevity. Thus, RR and PCA seem to promote mitochondrial turn-over, leading to the improved respiration that can be seen in our experiments.

Interestingly, even though positive effects can be seen on the respiratory chain system for RR and PCA, the mitochondrial membrane potential appears not to be affected by RR, but only PCA. Neither does RR show any improvements compared to M9 control, nor is it able to rescue the ΔΨm after an insult with PQ (see Figure 5c,d). The decreased potential after PQ insult is in line with the findings of Kanno et al. [81].

Here, ΔΨm represents the central parameter of bioenergetics that has a crucial influence on ATP synthesis in particular, as well as the generation of reactive oxygen species and the respiratory rate [82,83], but is not exclusively responsible for the respiratory rate [84]. A partial improvement of the respiratory chain after RR administration accompanied without an enhanced ΔΨm, therefore, seems plausible. These findings are coherent with the results of Hagl et al. on rice bran extract (RBE), which also improved, similar to our own findings, OXPHOS and complex IV, but again showed no effects on ΔΨm [49]. Concerning the effects of polyphenols on ΔΨm, growing evidence suggests a beneficial effect, but the literature is somewhat inconsistent. While Qi et al. report an improved membrane potential after treatment with tea polyphenols in SH-SY5Y cells [85] or an increased potential through resveratrol in rats with diabetic cardiomyopathy [86], Kumar et al. found a disrupted ΔΨm after prolonged exposure to resveratrol in murine prostate cancer cells [87]. 

PCA significantly improved the ΔΨm. This finding is in line with a study conducted by Liu et al., who found that PCA significantly attenuated PC12 cells stressed with rotenone in a dose dependent manner [88]. Apoptotic PC12 cells associated with mitochondrial dysfunction also showed an improved membrane potential after treatment with PCA [89].

Since partially increased respiration rates could be observed for both effectors and an improved membrane potential for PCA, we were interested in the consequential energetic output of the mitochondria in the form of ATP. Following the results of the ΔΨm experiments, RR had no significant influence on intracellular ATP levels, while concentrations were significantly increased by PCA **(**Figure 6). Mitochondrial membrane potential is the main driving force for mitochondrial energy production, thus it appears only feasible that improved ΔΨm leads to increased ATP levels [82,83]. Energy-enhancing capacities of already named polyphenols and numerous others have been well documented previously [90,91,92]. 

Beside mitochondrial ATP production, cells are able to produce energy via glycolysis [93]. Although glycolytic energy production is preferentially used in nematodes for cell growth and proliferation during larval stages L2–L4 [94,95], involvement of glycolysis in ATP generation has to be determined during adulthood to ensure the site of production. Increased lactate levels, through pyruvate conversion, is a marker for increased glycolytic activity [93]. Here, we show that RR significantly increased intracellular lactate levels, while PCA had no impact and pyruvate levels were not significantly altered by either effector. Consequently, no change in the lactate/pyruvate ratio could be observed for PCA but a significant increase was observed for RR, indicating an increased involvement of glycolysis during energy production. Mitochondrial impact on ATP synthesis is supposed to be more efficient than via glycolysis [96]. Therefore, it appears feasible that even though glycolytic activity appears to be increased by more than 2-fold, ATP levels are hardly affected after RR treatment, since ΔΨm is slightly but not significantly reduced. Concerning PCA, we suggest that increased respiratory chain activity is responsible for elevated ATP concentrations; lactate/pyruvate ratio is not altered, but ΔΨm is significantly improved. It has been well documented that polyphenols have an impact on oxidative phosphorylation, as well as glycolysis [97]. Curcumin and carnosol have proven to up-regulate glycolysis [98], as well as resveratrol [99] and caffeic acid [100], from which 3,4-dihydroxyhydrocinnamic acid is derived [101]. RR is rich in 3,4-dihydroxyhydrocinnamic acid, as our analysis shows. Although protocatechuic acid as a single substance appears to have no glycolysis altering capacity, RR, a composition of numerous polyphenols including 3,4-dihydroxyhydrocinnamic acid (see next paragraph), does.

### 4.3. Phenolic Compounds in RR

RR is an organic liquid concentrate made from fresh fruits, nuts, and vegetables, for example lemon, fig, dates, walnut, soybean, celery, and onion. This raw material was cascade fermented by different strains of *lactobacilli*. Therefore, the phenolic profile of RR is very complex. In total, 21 phenolic compounds, among them hydroxybenzoic acids, hydroxycinnamic acids, flavanones, flavones, and flavonols, were predominantly identified. During cascade fermentation, phenolic compounds undergo structural modification through bacterial enzymes and flavonoid aglycones were released from their corresponding glycosides by glycosidase activity [102]. As a result, the aglycones naringenin (**18**), hesperetin (**21**), luteolin (**16**), apigenin (**19**), quercetin (**17**), kaempferol (**20**), and daidzein (**15**) were present in RR and were released from their glycoconjugated flavonoids present in, for example, lemon, celery, onion, and soybean. The fragmentation pattern of the aglycones was in accordance with previously reported data [103]. These findings are in line with the phenolic profile described by Hippeli et al. (2007) [20]. In addition, rutinosides, such as hesperetin-7-*O*-rutinoside (**14**), eriodictyol-7-*O*-rutinoside (**11**), and kaempferol rutinoside (**12**), were tentatively identified. The release of such rutinosides by *lactobacilli* would involve α-l-rhamnosidase and β-glucosidase activity. Even if these enzymes were present during cascade fermentation, the pH needs to be adjusted to the optimal value to ensure high enzyme activity [104]. It is assumed that the pH value is greatly below the optimum, so that the enzymes were not able to catalyze the hydrolytic cleavage of rhamnose. In RR, the *C*-glycosides apigenin-6,8-di-*C*-glucoside (**7**) and two isomers of lucenin-2 methyl ether (**9**,**10**) were tentatively identified. Both compounds were reported in citrus juice [105]. This indicates that *C*-glycosides cannot be degraded by the *lactobacilli* strains used. There are two options to explain the occurrence of the hydroxybenzoic and hydroxycinnamic acids in the fermentation product. On the one hand, compounds such as gallic acid (**1**), protocatechuic acid (**2**), and *p*-coumaric acid (**8**) originated from walnut [106], for example. On the other hand, these compounds may be metabolites originating from bacterial degradation. Protocatechuic acid (**2**) is one of the most relevant fermentation metabolites. Its occurrence was confirmed in numerous food samples after microbial colonic fermentation in vitro and may be derived from red fruits (anthocyanins), chocolate (flavanols), orange juice (flavanones, anthocyanins), or apple juice (flavanols, flavonols) [107]. Furthermore, protocatechuic acid (**2**) was identified as a major human metabolite of cyanidin glycosides [108]. Apart from protocatechuic acid, it is possible that 3,4-dihydroxyhydrocinnamic acid (**6**) is derived from caffeic acid or chlorogenic acid [101], and 4-hydroxybenzoic acid (**5**) could be a metabolite of naringenin [109]. Gallic acid (**1**) and pyrogallol (**3**) are possible fermentation metabolites of tannins and gallic acid esters, as long as the *lactobacilli* strains contain enzymes, such as tannin acyl hydrolase [102].

The description of a fully analyzed fingerprint of polyphenolic compounds using a state-of–the-art method is a distinct advantage of our study. However, the lack of quantification is a limitation that must be addressed in further investigations. 

## 5. Conclusions

Fermentation of fruits and vegetables with *Lactobacillus rhamnosus* and *casei* produce metabolites of polyphenolic compounds. These metabolites promote molecular and cellular aspects of longevity, especially mitochondrial function, which might be beneficial for optimal health, and thus may contribute to healthy aging.

## Figures and Tables

**Table 1:** Oligonucleotide primer sequences, product sizes, and primer concentrations for quantitative real-time PCR.

Primer	Sequence	Product Size (bp)	Conc. (µM)
*ama-1*	5′-ccaggaacttcggctcagta-3′ 5′-tgtatgatggtgaagctggcg-3′	85	0.1
*act-2*	5′-cccactcaatccaaaggcta-3′ 5′-gggactgtgtgggraacacc-3′	168	0.1
*daf-16*	5′-tcctcattcactcccgattc-3′ 5′-ccggtgtattcatgaacgtg-3′	175	0.1
*sir-2.1*	5′-tggctgacgattcgatggat-3′ 5′-atgagcagaaatcgcgacac-3′	179	0.1
*skn-1*	5′-acagggtggaaaaagcaagg-3′ 5′-caggccaaacgccaatgac-3′	246	0.1

Note: bp = base pairs; Conc = concentration.

**Table 2:** LC-MS Identification of phenolic compounds in RR.

Peak	t_R_ (min)	UV_max_ (nm)	[M−H]^−^	[M+H]^+^	MS^n^ *m*/*z*	Compound
**1**	3.23	294	169		*MS*^2^: 125, 151 *MS*^3^[125]: 125, 81, 97	Gallic acid
**2**	5.37	260/295	153		*MS*^2^:109 *MS*^3^[109]: 109	Protocatechuic acid
**3**	5.81	276		127	*MS*^2^: 127, 109 *MS*^3^[127]: 127, 109	Pyrogallol
**4**	6.67	277	181		*MS*^2^: 163, 135 *MS*^3^[163]: 119	Dihydroxyhydrocinnamic acid
**5**	7.41	209/256	137		*MS*^2^: 93, 137 *MS*^3^[93]: 93	4-Hydroxybenzoic acid
**6**	8.60	282	181		*MS*^2^: 137, 163, 119, 109 *MS*^3^[137]: 137, 119, 109, 81, 93,95	3,4-Dihydroxyhydrocinnamic acid
**7**	11.47	272/331	593		*MS*^2^: 473, 503, 353, 575 *MS*^3^[473]: 353, 383	Apigenin 6,8-di-*C*-glucoside
**8**	11.71	218/311	163		*MS*^2^: 119, 163 *MS*^3^[119]: 119	*p*-Coumaric acid
**9**	12.04	272/349	623		*MS*^2^: 503, 383, 533, 413 *MS*^3^[503]: 383, 413	Lucenin-2 methyl ether Isomer 2
**10**	12.41	272/349	623		*MS*^2^: 503, 383, 533, 413 *MS*^3^[503]: 383, 413	Lucenin-2 methyl ether Isomer 1
**11**	13.81	285/330	595		*MS*^2^: 287 *MS*^3^[287]: 151	Eriodictyol-7-*O*-rutinoside
**12**	14.44	269/349	593		*MS*^2^: 285 *MS*^3^[285]: 285, 151	Kampferol rutinoside
**13**	16.46	260/349	447		*MS*^2^: 301, 300 *MS*^3^[301]: 179, 151, 273	Quercetin rhamnoside
**14**	16.63	285	609		*MS*^2^: 301 *MS*^3^[301]: 286, 242, 283, 257, 125	Hesperetin-7-*O*-rutinoside
**15**	18.70	304	253		*MS*^2^: 253 *MS*^3^[253]: 253	Daidzein
**16**	19.68	372 ^A^	285		*MS*^2^: 285, 241, 217, 199, 175, 257, 151 *MS*^3^[285]: 241, 243, 213	Luteolin
**17**	19.68	372	301		*MS*^2^: 179, 151, 257, 273 *MS*^3^[179]: 151, 179	Quercetin
**18**	20.67	285/330 sh	271		*MS*^2^: 151, 177, 165, 107 *MS*^3^[151]: 107, 151	Naringenin
**19**	20.82	261/330	269		*MS*^2^: 269, 201, 181, 225, 169 *MS*^3^[269]: 269	Apigenin
**20**	21.03	363	285		*MS*^2^: 285, 151 *MS*^3^[285]: 285	Kaempferol
**21**	21.17	288/330 sh	301		*MS*^2^: 286, 242, 283 257, 125, 199, 151 *MS*^3^[286]: 258, 242, 199, 174, 268, 215	Hesperetin

Note: ^A^ Coelution with Quercetin; sh = shoulder; MS^n^ fragment ions are listed in order of decreasing intensity; precursors for MS^3^ in brackets; t_R_ (min) = retention time in minutes, UV_max_ (nm) = peak maximum detected in ultraviolet light in nanometer; *m*/*z* = mass to charge ratio; [M−H]^−^ = negative molecular ion; [M−H]^+^ = positive molecular ion.
